# Circumferential Repair Versus Labral Base Refixation for the Treatment of Symptomatic Femoroacetabular Impingement Syndrome: A Systematic Review and Narrative Synthesis

**DOI:** 10.1177/23259671251389140

**Published:** 2025-12-19

**Authors:** Matey Juric, Koorosh Kashanian, Darius L. Lameire, Hassaan Abdel Khalik, Audrey Champagne, Tim Dwyer, Daniel B. Whelan, Jaskarndip Chahal

**Affiliations:** † Faculty of Medicine, University of Ottawa, Ottawa, Ontario, Canada; ‡ Division of Orthopaedic Surgery, University of Alberta, Edmonton, Alberta, Canada; § Division of Orthopaedic Surgery, University of Toronto, Toronto, Ontario, Canada; ‖ Division of Orthopaedic Surgery, McMaster University, Hamilton, Ontario, Canada; ¶ University of Toronto Orthopaedic Sports Medicine, Toronto, Ontario, Canada; # Division of Orthopaedic Surgery, Mount Sinai Hospital, Toronto, Ontario, Canada; ** Division of Orthopaedic Surgery, St Michael's Hospital, Toronto, Ontario, Canada; ‡‡ Division of Orthopaedic Surgery, Women's College Hospital, Toronto, Ontario, Canada; Investigation performed at the University of Toronto, Toronto, Ontario, Canada

**Keywords:** hip arthroscopy, labral repair, suture techniques, systematic review

## Abstract

**Background:**

Hip arthroscopy is a common surgical treatment method for femoroacetabular impingement syndrome (FAIS) and typically involves labral repair. Suture limbs can either be placed around (circumferential repair technique) or through (labral base refixation [LBR] technique) the labrum; however, there remains a lack of consensus regarding the superiority of either technique.

**Purpose:**

To evaluate and narratively synthesize the available evidence on patient-reported outcome measure (PROM) scores of LBR and circumferential repair in patients undergoing hip arthroscopy and labral repair for FAIS.

**Study Design:**

Systematic review; Level of evidence, 4.

**Methods:**

A systematic electronic search of MEDLINE, Embase, and the Cochrane Library was carried out on July 21, 2024. All English-language randomized controlled trials, comparative studies, and case series on adults with symptomatic FAIS were eligible for inclusion.

**Results:**

A total of 12 studies with 1488 patients were included in the analysis. Overall, 9 cohorts with 1035 patients and a mean age of 33.1 years were included in the circumferential repair group, and 6 cohorts with 453 patients and a mean age of 32.3 years were included in the LBR group.Both the circumferential repair and LBR techniques were associated with significant improvements on PROMs, including the mHHS (modified Harris Hip Score), HOS-ADL (Hip Outcome Score–Activities of Daily Living), HOS-SSS (Hip Outcome Score–Sports-Specific Subscale), NAHS (Non-Arthritic Hip Score), WOMAC (Western Ontario and McMaster Universities Osteoarthritis Index), and VAS (visual analog scale). Postoperative scores were commonly >80 points across measures, with mean improvements of 20 to 30 points in function and 2 to 4 points in pain. Reported rates of revision surgery and conversion to total hip arthroplasty were low across both techniques, generally <10%, although some variability existed between studies.

**Conclusion:**

Both techniques led to improved PROM scores after hip arthroscopy for the management of FAIS. Given the heterogeneity and predominance of lower level evidence, future high-quality comparative studies are warranted.

Femoroacetabular impingement syndrome (FAIS) is a painful condition in which there is abnormal contact between the femoral head and the acetabulum.^3^ It typically develops from anatomic abnormalities such as cam lesions (aspherical femoral head) or pincer lesions (overcoverage of the acetabulum), which can lead to repetitive microtrauma and progressive damage to the labrum and cartilage.^14^ Clinically, patients often have groin pain that worsens with activity, reduced range of motion, and a positive impingement test finding (pain with flexion, abduction, and internal rotation).^33^ Biomechanically, altered joint congruency leads to increased shear forces across the chondrolabral junction, which can compromise stability and predispose patients to early osteoarthritic changes.^4^ Hip arthroscopy for the treatment of FAIS often includes femoroplasty or acetabuloplasty, and for a torn labrum, labral debridement, repair, or reconstruction is commonly performed.^5,15^

The labrum is a strong fibrocartilaginous ring around the rim of the acetabulum that forms a suction seal between the femoral head and the hip socket. The suction seal plays an important role in increasing stability of the hip, as it reduces pressure and friction in the joint, which helps to prevent wear on cartilage and potentially lowers the risk of developing osteoarthritis.^4,35^ Therefore, fixing a torn labrum is an important step when aiming to restore patients’ function and decrease pain. Currently, several arthroscopic approaches for a torn labrum exist, with the 3 most common being labral debridement, repair, and reconstruction. While several studies have shown that labral repair is associated with improved patient-reported outcome measure (PROM) scores and lower rates of conversion to total hip arthroplasty (THA),^17,23^ others have demonstrated that labral debridement can yield comparable clinical outcomes, especially in cases in which the labrum is irreparable or degenerative.^9,24^ This highlights the importance of individualized surgical decision making in which both preservation and selective debridement play valuable roles, depending on intraoperative findings and patient characteristics.

Repair of the labrum can be accomplished through either a circumferential repair or labral base refixation (LBR) technique. With the circumferential repair technique, one limb of the suture passes between the labrum and the acetabular rim and is retrieved over the top of the labrum, creating a loop.The LBR technique consists of passing one limb between the labrum and the acetabular rim, followed by pushing a suture passer through the midsubstance of the labrum and retrieving back the limb through the labrum.^37^ Unlike the circumferential repair technique, which may lead to nonanatomic eversion of the labrum, the LBR technique attempts to invert tissue to re-establish the seal,^13^ which can restore stability to the joint while preventing further chondral damage or the progression of osteoarthritis. However, the latter approach can lead to splitting or possibly tearing of the labrum.^7,40^ A previous biomechanical study looked at the ability of these 2 techniques to restore the suction seal effect in a cadaveric model by measuring intra-articular hip fluid pressurization in the labrum and found that a combination of the 2 techniques most closely replicated the labrum in its intact state.^34^ However, a better understanding is needed in human participants.

Previous comparative studies have aimed to establish whether one suture technique is superior to the other; however, there is inconsistency in the findings, with some studies reporting no significant difference between the 2 techniques and others finding improvements with one technique versus another.^29,41^ However, there are no reviews that have compared these 2 techniques for the treatment of FAIS and have investigated which suture method results in superior outcomes. Therefore, this systematic review aimed to narratively synthesize the outcomes associated with the circumferential repair and LBR techniques used during hip arthroscopy for FAIS. Rather than attempting to determine superiority, we sought to describe outcome trends across studies to inform surgical decision-making and highlight areas for future research.

## Methods

This systematic review followed the algorithm and guidelines recommended by the Preferred Reporting Items for Systematic Reviews and Meta-Analyses (PRISMA).^31^ The protocol was registered with the International Prospective Register of Systematic Reviews (PROSPERO) following the PRISMA-P guidelines (No. 575316).

### Comprehensive Search Strategy

A systematic electronic search of 3 databases (MEDLINE, Embase, and CENTRAL [Cochrane Central Register of Controlled Trials]) was performed through July 21, 2024, by 3 reviewers (M.J., K.K., and D.L.L.) for literature related to labral repair for FAIS since database inception. Search items included “hip,”“arthroscopy,” and “labrum,” among several others. The full search strategy used for each database can be found in Appendix Table A1. Inclusion criteria for articles included (1) adult patients (aged >18 years); (2) symptomatic FAIS; (3) hip arthroscopy with concomitant labral repair (circumferential repair or LBR); and (4) randomized controlled trials, comparative studies, or case series. Exclusion criteria consisted of (1) labral reconstruction, (2) studies not available in English, (3) diagnostic hip arthroscopy, (4) revision arthroscopy, (5) previous surgery or infections (if reported), (6) hip dysplasia, (7) rheumatoid arthritis, and (8) concomitant periacetabular osteotomy.

### Study Screening

**Figure 1.**
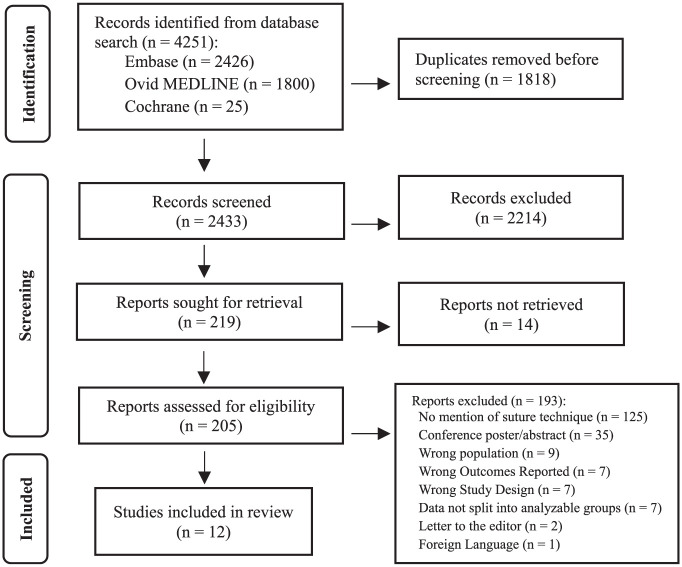
PRISMA (Preferred Reporting Items for Systematic Reviews and Meta-Analyses) flow diagram.

There were 2 authors (M.J. and K.K.) who independently screened the titles and abstracts of all identified studies, removing duplicates (Figure 1). All titles and abstracts were screened independently, and all conflicts progressed to a full-text review. All included studies were assessed by a full-text review, and any disagreement was reassessed by both reviewers independently, while any articles that did not attain agreement after this stage were reviewed by a third author (D.L.L.). A kappa value was calculated for the title/abstract and full-text screening stages to determine the level of agreement between reviewers. Based on previous studies, kappa values were defined a priori: <0.00, no agreement; 0.00-0.19, some agreement; 0.20-0.39, fair agreement; 0.40-0.59, moderate agreement; 0.60-0.79, substantial agreement; and 0.80-0.99, near perfect agreement.^30^

The Methodological Index for Non-Randomized Studies (MINORS) criteria were used to assess each study's risk of bias. They include 12 questions for comparative studies and 8 questions for noncomparative studies, which were rated as follows: 0, not reported; 1, reported but inadequate; and 2, reported and adequate. The maximum scores are 24 and 16 for comparative studies and noncomparative studies, respectively. Comparative studies with scores ≤14, 15-22, and 23-24 were deemed poor, moderate, and good quality, respectively. For noncomparative studies, a score ≤8 was considered poor quality, 9-14 was considered moderate quality, and 15-16 was considered good quality.^39^ All studies were reviewed by 2 authors (M.J. and K.K.), with disagreements resolved by discussions.

### Data Collection

There were 3 reviewers (M.J., K.K., and D.L.L.) who were involved in data collection; 2 reviewers (M.J. and K.K.) obtained data from half the studies each, while the third reviewer appraised accuracy. The data were recorded into tables using Google Sheets (Google). The following data were collected from studies if available: study characteristics (author[s], year, title, journal, recruitment period, country, study design, level of evidence, inclusion and exclusion criteria), intervention groups, number of patients, follow-up length, participant characteristics (age, sex, laterality, type of FAIS), intraoperative findings (Seldes classification, traction time, acetabular Outerbridge grade, femoral head Outerbridge grade, ligamentum teres tears), concomitant procedures as demonstrated in Table 1 (debridement, capsular repair, iliopsoas release, rim trimming, chondroplasty, osteoplasty, trochanteric bursectomy, acetabuloplasty, femoroplasty, selective debridement of ligamentum teres, microfracture), PROM scores (modified Harris Hip Score [mHHS], Hip Outcome Score–Activities of Daily Living [HOS-ADL], Hip Outcome Score–Sports-Specific Subscale [HOS-SSS], Non-Arthritic Hip Score [NAHS], visual analog scale [VAS], patient satisfaction, Western Ontario and McMaster Universities Osteoarthritis Index [WOMAC], 36-item Short Form Health Survey [SF-36], University of California, Los Angeles [UCLA] activity scale), reoperations, and revisions. The level of evidence of each study was classified based on the authors’ statements.

**Table 1:** Concomitant Procedures Performed

	n (%)
Circumferential repair (n = 1056)	
Capsular repair	315 (29.11)
Iliopsoas release	58 (5.36)
Rim trimming	290 (26.80)
Chondroplasty	0 (0.00)
Osteoplasty	66 (6.10)
Trochanteric bursectomy	3 (0.28)
Acetabuloplasty	488 (45.10)
Femoroplasty	420 (38.82)
Selective debridement of ligamentum teres	86 (7.95)
Microfracture	53 (4.90)
Total	1779
Labral base refixation (n = 461)	
Capsular repair	119 (25.81)
Iliopsoas release	67 (14.53)
Chondroplasty	68 (14.75)
Osteoplasty	98 (21.26)
Trochanteric bursectomy	9 (1.95)
Acetabuloplasty	202 (43.82)
Femoroplasty	44 (9.54)
Selective debridement of ligamentum teres	66 (14.32)
Microfracture	14 (3.04)
Total	687

### Outcomes

Results were narratively synthesized by describing trends in PROM scores and complications across individual studies. The secondary outcomes synthesized for this study were the risks of conversion to THA and revision surgery of any type.

### Statistical Analysis

Descriptive statistics were used to summarize the data, which included calculating means, percentages, and ranges using Google Sheets. Because of the heterogeneity of study design, follow-up duration, and outcome reporting, no statistical comparisons or pooled analyses were conducted. Instead, outcome trends were synthesized narratively across studies. Additionally, kappa values with their standard deviations and 95% confidence intervals were calculated.

## Results

### Study Characteristics

The search strategy resulted in 2433 articles after the removal of duplicates, with 8 (66.7%) case series, 2 (16.7%) comparative studies, and 2 (16.7%) prospective cohort studies included in the final analysis (Table 2).^2,6,8-10,18-20,25,28,29,37^ Of the 12 included studies, 6 were single-arm studies that looked at the circumferential repair technique, 3 were single-arm studies assessing the LBR technique, and 3 studies compared circumferential repair to LBR, yielding 9 cohorts for the circumferential repair technique and 6 cohorts for the LBR technique (Appendix Table A2). Among the included studies, 1 (8.3%) was classified as having a level of evidence of 2, four (33.3%) were classified as having a level of evidence of 3, and 7 (58.3%) were classified as having a level of evidence of 4. Publication dates of all included articles were within 10 years of the literature search date. The reviewers found both comparative and noncomparative studies to be of moderate quality using the MINORS criteria in which the mean score from both reviewers was 16.4 ± 1.8 for comparative studies and 10.8 ± 1.0 versus 11.0 ± 0.8 for noncomparative studies.

**Table 2:** Patient Characteristics^*a*^

							FAIS Type, %		
First Author (Year)	No. of Patients	No. of Hips	Female Sex, %	Age,^*b*^ y	Follow-up,^*b*^ y	Side, R/L, n	Cam	Pincer	Mixed
Circumferential repair									
Avnieli^2^ (2020)									
NWB	69	69	36.23	35.8 ± 14.3	2 (minimum)	NR	NR	NR	NR
WB	64	64	40.63	32.9 ± 14.5	2 (minimum)	NR	NR	NR	NR
Byrd^6^ (2014)	37	38	71.05	26 ± NR (11-44)	2 ± NR	20/18	28.95	NR	NR
Carton^8^ (2018)	104	123	15.38	26.7 ± NR (19-49)	2.4 ± NR (1.7-3.2)	NR	NR	NR	66.7
Cetinkaya^9^ (2016)	33	34	55.88	33.5 ± NR (30-61)	3.8 ± NR (2.4-5.4)	NR	2.94	52.94	44.12
Jackson^18^ (2015)	110	110	69.09	27.43 ± NR	2.45 ± NR (1.60-5.58)	55/55	NR	NR	NR
Lee^25^ (2019)	41	41	48.78	34.6 ± NR (16-54)	7.7 ± NR (7.1-9.8)	26/15	NR	NR	NR
Maldonado^28^ (2020)	309	309	68.28	36.2 ± 14.5	2.54 ± 0.53	157/152	NR	NR	NR
May^29^ (2020)	79	79	40.00	32.79 ± NR	1.32 ± NR	NR	NR	NR	NR
Sawyer^37^ (2015)	189	189	48.15	35.9 ± 11.3	3.3 ± 0.9	NR	2.65	1.06	96.3
Labral base refixation									
Domb^10^ (2017)	60	64	73.44	28.9 ± 11.8	5.65 ± 0.61 (5.00-7.48)	29/35	NR	NR	NR
Jackson^19^ (2014)	54	54	62.96	28.8 ± 12.8 (14-57)	2.4 ± 0.6 (1.7-4.1)	28/26	5.50	35.0	59.5
Jackson^18^ (2015)	110	110	69.09	27.3 ± NR	2.5 ± NR (1.6-5.0)	53/57	NR	NR	NR
Kaplan^20^ (2021)	103	107	68.22	39.4 ± 12.4	6.38 ± 1.59 (2.50-9.42)	55/52	NR	NR	NR
May^29^ (2020)	66	66	40.00	32.79 ± NR	1.32 ± NR	NR	NR	NR	NR
Sawyer^37^ (2015)	60	60	48.33	35.9 ± 11.3	3.07 ± 0.74	NR	NR	3.33	96.67

a FAIS, femoroacetabular impingement syndrome; L, left; NR, not reported; NWB, nonweightbearing; R, right; WB, weightbearing.

b Data are shown as mean ± SD or mean ± SD (range) unless otherwise indicated.

The 12 included studies consisted of 1488 patients and 1517 hips with a mean age of 32.9 years and a mean follow-up of 3.12 years. Across all studies, the proportion of female patients was 54.7%. The circumferential repair group consisted of 1035 patients (1056 hips) with a mean age of 33.1 years and a weighted mean follow-up of 2.81 years, and the proportion of female patients was 51.6%. In the LBR group, 453 patients (461 hips) with a mean age of 32.3 years were included; the weighted mean follow-up was 3.73 years, and the proportion of female patients was 61.9% (Table 2).

### PROM Scores

The most reported PROMs used among the included studies were the mHHS, HOS-ADL, HOS-SSS, NAHS, WOMAC, and VAS (Table 3). Across the studies, both the circumferential repair and LBR techniques were associated with consistent improvements on all reported PROMs. For the mHHS, postoperative scores of the circumferential repair group ranged from 83 to 100, with most studies reporting improvements ≥20 points from preoperatively. Similarly, studies on the LBR technique also demonstrated substantial improvements, with postoperative scores ranging between 83 and 90. The HOS-ADL and HOS-SSS followed similar trends, with most postoperative scores >80 and mean improvements of 20 to 30 points. The NAHS also showed marked improvements across both techniques, increasing from preoperative mean scores from 51 to 64 to postoperative scores between 84 and 88. WOMAC scores improved with postoperative decreases, indicating reduced pain and stiffness. VAS scores consistently dropped by 2 to 4 points, suggesting clinically meaningful reductions in pain. Although variations in preoperative scores and follow-up duration existed, the overall pattern across individual studies supports functional and symptomatic improvements after either labral repair technique (Table 3).

**Table 3:** Patient-Reported Outcome Measure Scores^*a*^

First Author (Year)	No. of Hips	Preoperative	Postoperative	*P*
mHHS				
Circumferential repair				
Avnieli^2^ (2020)				
NWB	69	62.1 ± NR (57-66)	84.5 ± NR (79-89)	NR
WB	64	65.1 ± NR (59-69)	86.7 ± NR (78-89)	NR
Byrd^6^ (2014)	38	70.5 ± NR	89.4 ± NR	<.0001
Carton^8^ (2018)	123	76 ± NR (70-88)	100 ± NR (96-100)	.001
Jackson^18^ (2015)	110	64.0 ± 3.1	86.0 ± 2.7	<.0001
Lee^25^ (2019)	41	59.5 ± NR (37.5-82.0)	86.8 ± NR (61.0-95.7)	<.001
Maldonado^28^ (2020)	309	62.6 ± 15.7 (60.9-64.4)	86.9 ± 16.2 (85.1-88.7)	<.001
Sawyer^37^ (2015)	189	63.9 ± 14.6	82.7 ± 14.7	NR
Labral base refixation				
Domb^10^ (2017)	64	64.4 ± 13.8	85.3 ± 17.7	<.001
Jackson^19^ (2014)	54	63.7 ± 17.9	89.9 ± 13.0	<.00001
Jackson^18^ (2015)	110	64.0 ± 2.6	84.0 ± 3.0	<.0001
Kaplan^20^ (2021)	107	48.4 ± NR	84.4 ± NR	NR
Sawyer^37^ (2015)	60	61.9 ± 14.1	83.0 ± 14.2	NR
HOS-ADL				
Circumferential repair				
Avnieli^2^ (2020)				
NWB	69	67.2 ± NR (62-72)	83.1 ± NR (78-88)	NR
WB	64	63.7 ± NR (52-70)	88.4 ± NR (80-90)	NR
Cetinkaya^9^ (2016)	34	55.12 ± 5.98 (40-62)	87.18 ± 11.30 (50-99)	<.05
Jackson^18^ (2015)	110	67.0 ± 3.5	88.0 ± 2.8	<.0001
Lee^25^ (2019)	41	58.3 ± NR (20-80)	85.2 ± NR (45-95)	<.001
Sawyer^37^ (2015)	189	68.1 ± 19.4	90.7 ± 11.3	NR
Labral base refixation				
Jackson^19^ (2014)	54	66.9 ± 21.2	91.0 ± 14.4	<.00001
Jackson^18^ (2015)	110	66.0 ± 3.5	87 ± 3	<.0001
Sawyer^37^ (2015)	60	63.8 ± 18.8	88.7 ± 15.6	NR
HOS-SSS				
Circumferential repair				
Jackson^18^ (2015)	110	45.0 ± 4.3	76.0 ± 5.2	<.0001
Lee^25^ (2019)	41	51.2 ± NR (10-80)	82.4 ± NR (50-95)	<.001
Maldonado^28^ (2020)	309	39.8 ± 22.0 (37.3-42.3)	74.2 ± 27.3 (71.1-77.2)	<.001
Sawyer^37^ (2015)	189	50.6 ± 25.3	81.0 ± 20.8	.479
Labral base refixation				
Domb^10^ (2017)	64	47.1 ± 23.2	76.5 ± 25.9	<.001
Jackson^19^ (2014)	54	46.5 ± 24.8	79.2 ± 25.4	<.00001
Jackson^18^ (2015)	110	46.0 ± 4.4	76.0 ± 4.7	<.0001
Sawyer^37^ (2015)	60	46.4 ± 22.6	77.1 ± 26.6	.479
NAHS				
Circumferential repair				
Jackson^18^ (2015)	110	63.0 ± 3.5	86.0 ± 2.5	<.0001
Maldonado^28^ (2020)	309	63.1 ± 16.7 (64.9-61.2)	86.1 ± 16.7 (84.2-88.0)	<.001
May^29^ (2020)	79	10.3 ± NR	14.7 ± NR	NR
Labral base refixation				
Domb^10^ (2017)	64	63.7 ± 17.0	87.0 ± 14.7	<.001
Jackson^19^ (2014)	54	60.9 ± 21.1	87.9 ± 15.7	<.00001
Jackson^18^ (2015)	110	61.0 ± 3.5	84.0 ± 3.3	<.0001
Kaplan^20^ (2021)	107	51.2 ± NR	85.8 ± NR	NR
May^29^ (2020)	66	9.6 ± NR	17.7 ± NR	NR
WOMAC				
Circumferential repair				
Carton^8^ (2018)	123	18 ± NR (8-31)	1 ± NR (0-4)	<.001
Sawyer^37^ (2015)	189	22.6 ± 15.1	9.2 ± 10.1	NR
Labral base refixation				
Sawyer^37^ (2015)	60	34.2 ± 18.3	11.9 ± 15.3	NR
VAS				
Circumferential repair				
Cetinkaya^9^ (2016)	34	8.0 ± NR (6.8-9.0)	2.3 ± NR (0-3)	NR
Jackson^18^ (2015)	110	6.0 ± 0.4	2.47 ± 0.47	<.0001
Lee^25^ (2019)	41	6.4 ± NR (2-9)	1.8 ± NR (0-7)	<.001
Maldonado^28^ (2020)	309	5.0 ± 2.3 (4.7-5.2)	2.0 ± 2.4 (1.7-2.2)	<.001
Labral base refixation				
Domb^10^ (2017)	64	5.9 ± 2.4	2.0 ± 2.1	<.001
Jackson^19^ (2014)	54	6.5 ± 2.2	2.3 ± 2.3	<.00001

a Data are shown as mean ± SD or mean ± SD (range). HOS-ADL, Hip Outcome Score–Activities of Daily Living; HOS-SSS, Hip Outcome Score–Sports-Specific Subscale; mHHS, modified Harris Hip Score; NAHS, Non-Arthritic Hip Score; NR, not reported; NWB, nonweightbearing; VAS, visual analog scale; WB, weightbearing; WOMAC, Western Ontario and McMaster Universities Osteoarthritis Index.

### Reoperations

Both the circumferential repair and LBR techniques were associated with relatively low rates of conversion to THA and revision arthroscopy. Among studies using circumferential repair, THA conversion rates ranged from 0.0% to 5.9%, with most studies reporting rates <3%. For LBR, Domb etal^10^ reported the highest THA conversion rate at 9.4%, while multiple studies reported rates <1% (Table 4).

**Table 4:** THA Conversions and Reoperations^*a*^

		Conversion to THA			Revision Hip Arthroscopy		
First Author (Year)	No. of Hips	n (%)	Age,^*b*^ y	Time to THA,^*c*^ mo	n (%)	Age,^*b*^ y	Time to Revision,^*c*^ mo
Circumferential repair							
Avnieli^2^ (2020)							
NWB	69	0 (0.00)	NR	NR	0 (0.00)	NR	NR
WB	64	0 (0.00)	NR	NR	0 (0.00)	NR	NR
Byrd^6^ (2014)	38	NR	NR	NR	4 (10.53)	NR	10 ± NR (5-15)
Carton^8^ (2018)	123	0 (0.00)	NR	NR	5 (4.07)	NR	NR
Cetinkaya^9^ (2016)	34	2 (5.88)	NR	16 ± NR (8-32)	1 (2.94)	NR	NR
Jackson^18^ (2015)	110	2 (1.82)	NR	NR	10 (9.09)	NR	NR
Lee^25^ (2019)	41	1 (2.44)	NR	86 ± NR	5 (12.20)	NR	26.6 ± NR (15-49)
Maldonado^28^ (2020)	309	8 (2.59)	NR	NR	21 (6.80)	NR	NR
May^29^ (2020)	79	NR	NR	NR	NR	NR	NR
Sawyer^37^ (2015)	189	6 (3.17)	46-57	NR	14 (7.41)	NR	NR
Labral base refixation							
Domb^10^ (2017)	64	6 (9.38)	NR	45.2 ± 30.7 (10.1-83.4)	11 (17.19)	NR	23.3 ± 13.7 (7.9-49.7)
Jackson^19^ (2014)	54	2 (3.70)	50.5	18 ± NR	3 (5.56)	NR	22.26 ± NR (10.50-34.45)
Jackson^18^ (2015)	110	1 (0.91)	NR	NR	10 (9.09)	NR	NR
Kaplan^20^ (2021)	107	1 (0.93)	NR	NR	3 (2.80)	NR	NR
May^29^ (2020)	66	NR	NR	NR	NR	NR	NR
Sawyer^37^ (2015)	60	0 (0.00)	NR	NR	5 (8.33)	NR	NR

a NR, not reported; NWB, nonweightbearing; THA, total hip arthroplasty; WB, weightbearing.

b Data are shown as mean or range.

c Data are shown as mean ± SD or mean ± SD (range).

Revision arthroscopy was similarly infrequent across both techniques. In circumferential repair studies, revision rates ranged from 0.0% to 12.2%. In studies evaluating LBR, revision rates ranged from 0.0% to 17.2%, with the highest rate reported by Domb etal.^10^ The mean time to revision was variably reported but generally ranged between 1 and 2 years postoperatively when available. Overall, while reoperations and THA conversions occurred in both groups, rates were low and comparable and may reflect differences in follow-up duration, patient selection, and surgeon thresholds for a reintervention (Table 4).

## Discussion

The primary finding of this systematic review is that both circumferential repair and LBR resulted in postoperative improvements in PROM scores and that overall either technique was effective in repairing the labrum. Among the PROMs evaluated, the mHHS and HOS-ADL were the most consistently reported and meaningful in patients undergoing labral repair for FAIS. Both measures demonstrated substantial postoperative improvements across the included studies and captured aspects of both function and daily activity, which are relevant to this population. As such, the mHHS and HOS-ADL provided the most reliable insight into functional recovery after labral repair for the population in the current study.

While there is evidence of superior outcomes after repair of the labrum compared with labral debridement when arthroscopically treating FAIS,^1,12,24,32^ there lacks a consensus about the optimal suture technique for repair.^19,29,37^ It is thought that LBR allows the surgeon to preserve the hip joint's suction seal by only incorporating the base of the labrum in the repair construct, ultimately allowing the labrum's free edge to be in contact with the femoral head without interruption.^18,19^ While LBR maximizes the contact area between the labrum and femoral head, it can be technically challenging in certain scenarios. For example, a hypotrophic labrum (generally <4 mm) does not provide enough tissue for a translabral suture and ultimately requires the circumferential repair technique.^11,29^ In this systematic review, 3 studies^10,19,29^ mentioned how the thickness of labral tissue influenced the decision to use the circumferential repair or LBR technique. Another notable downside of this technique is that it may cause further iatrogenic tears secondary to piercing the labrum. However, Fry and Domb^13^ stated that the use of a small-diameter suture-passing device in labra that are >3 mm in width can circumvent this problem.

Circumferential sutures may be warranted in labra that have intrasubstance damage, which are tears involving the labral body, or in instances in which the labrum is hypotrophic such as in pincer lesions.^27,29^ Often, the circumferential repair technique has been criticized in the literature for bunching the labrum or distorting its normal triangular cross-sectional anatomy, which prevents it from contacting the femoral head.^13^ To counter this criticism, Lertwanich etal^26^ performed magnetic resonance imaging at 3 weeks after hip arthroscopy and demonstrated that the triangular cross-sectional anatomy of the labrum was not distorted in patients using the circumferential repair technique. In general, this technique requires looping a suture across the entire labrum, which is likely to lead to lifting the labrum onto the acetabular rim and off the femoral head, reducing the suction seal effect.^19^ To support this claim, a cadaveric study showed that circumferential sutures do not restore the normal sealing properties of the labrum^7^; however, there are several studies that have demonstrated good clinical outcomes with this technique.^21,24,32,36-38^ Additionally, a comparative study showed that surgeons felt that they were able to effectively restore the suction seal effect of the labrum using this technique.^18^ The location of the labral tear may also have an effect on outcomes; however, none of the included studies commented on the location of the tear using clock-face nomenclature.

Although both techniques have strengths and weaknesses, it is incredibly difficult to elucidate which, if any, is the superior technique. Both have demonstrated positive results regarding PROMs; however, when directly comparing the 2 techniques, it may be difficult to account for all confounding variables, which may skew results. For example, concomitant procedures such as femoral or acetabular osteochondroplasty, as listed in Table 1, are commonly performed for the treatment of cam and pincer lesions, which improve a patient's range of motion and could ultimately affect clinical outcomes.^25^ However, the included studies did not stratify outcomes by concomitant procedures, making it difficult to know the true effect that they may have had on PROM scores. Additionally, the type of suture anchor (knotted or knotless), as well as the number used,may vary from patient to patient and is not always reported in the literature. In the included studies, 7 of 12 studies detailed the type of suture anchor used,^9,10,18-20,28,37^ while only 2 of 12 studies reported on the mean number of anchors used,^9,18^ which may have an effect on outcomes.^9,18^ Another potential confounding variable that could have played a role in patient outcomes is adherence to rehabilitation protocols postoperatively. Rehabilitation and early joint mobilization are thought to be essential in ensuring proper recovery and the prevention of intra-articular adhesions.^16,22^ While 7 of the included studies explicitly reported their rehabilitation protocols, none stated how adherent patients were with them at follow-up.^2,6,9,10,20,28,29^ However, given that none of the reported PROM scores between the 2 intervention groups were drastically different from one another, it is less likely that adherence to rehabilitation programs had a significant effect.

Overall, the findings of the present study indicate that both techniques showed improvements in outcomes, as measured by PROMs and revision rates. Future studies should aim to increase homogeneity among their patients by controlling for variables such as concomitant procedures and the number of suture anchors used, among others. Additionally, future studies should aim to randomize patients between the 2 techniques and assess the restoration of the suction seal at time zero in the operating room.

### Limitations

The included studies showed significant heterogeneity of patients (ie, number of patients with cam vs pincer lesions) as well as the study design and PROMs. Using PROM scores as the primary outcome may skew results because of the subjective reporting of patients. Furthermore, this review included studies mainly of level 3 and 4 evidence and lacked randomized controlled trials, increasing the potential for bias. The cartilage score can also have an effect on outcomes; however, it was not possible to stratify outcomes based on the cartilage grade, given the paucity of cartilage grades reported in the studies. Additionally, the potential for overlapping patient populations across studies is a notable limitation. Multiple studies by the same authors or institutions, particularly the 2 studies by Jackson etal^18,19^ and that from Domb etal,^10^ may include the same or partially overlapping cohorts. Further, the primary purpose for most of the included studies was not to assess a suture technique; as such, the number of sutures used could have been variable across studies, further impacting outcomes. This may introduce significant bias to the outcomes; however, given the relative paucity of data specifically comparing the 2 suturing techniques, this review could be considered a baseline understanding for which future trials can be based on.

## Conclusion

Both circumferential repair and LBR are effective techniques that led to improved PROM scores after hip arthroscopy for the management of FAIS. Additionally, both techniques showed low and comparable rates of revision surgery and conversion to THA. Future research should focus on high-quality randomized controlled trials that control for confounding variables, assess the restoration of the suction seal intraoperatively, and include long-term follow-up.Such studies are essential to establish more definitive guidance on technique selection and optimize outcomes in patients undergoing hip arthroscopy for FAIS.

## Appendix

**Table A1:** Search Strategy

MEDLINE and Embase	Cochrane Library
(1) hip.mp.	(1) hip
(2) femoroacet*.mp.	(2) femur
(3) exp Hip Injuries/ or exp Hip/	(3) femo*
(4) 1 or 2 or 3	(4) FAI
(5) arthrosco*.mp.	(5) 1 or 2 or 3 or 4
(6) exp Arthroscopy/	(6) arthroscopy
(7) 5 or 6	(7) scope
(8) random*.mp.	(8) osteochondroplasty
(9) exp Randomized Controlled Trial/	(9) labral
(10) prospect*.mp.	(10) 6 or 7 or 8 or 9
(11) 8 or 9 or 10	(11) 5 and 10
(12) 4 and 7 and 11	

**Table A2:** Study Characteristics^*a*^

First Author (Year)	Journal	Recruitment Period	Country	Study Design	LOE	Group 1	Group 2
Avnieli^2^ (2020)	*Arthroscopy*	Jan 2011–Jun 2016	Israel	Case series	3	Circumferential repair (NWB)	Circumferential repair (WB)
Byrd^6^ (2014)	*Arthroscopy*	2007-2010	USA	Case series	4	Circumferential repair	
Carton^8^ (2018)	*J Hip Preserv Surg*	Nov 2013–Aug 2014	Ireland	Case series	4	Circumferential repair	
Cetinkaya^9^ (2016)	*Hip Int*	Jul 2008–Dec 2011	Turkey	Comparative study	3	Circumferential repair	
Domb^10^ (2017)	*Am J Sports Med*	Feb 2008–May 2011	USA	Case series	4	LBR	
Jackson^19^ (2014)	*Arthroscopy*	Apr 2008–Nov 2010	USA	Case series	4	LBR	
Jackson^18^ (2015)	*Arthroscopy*	Feb 2008–Feb 2012	USA	Comparative study	3	Circumferential repair	LBR
Kaplan^20^ (2021)	*Arthroscopy*	2010-2017	USA	Case series	4	LBR	
Lee^25^ (2019)	*Clin Orthop Surg*	Jan 2008–Dec 2010	Republic of Korea	Case series	4	Circumferential repair	
Maldonado^28^ (2020)	*Orthop J Sports Med*	Feb 2015–Jan 2017	USA	Case series	4	Circumferential repair	
May^29^ (2020)	*Orthop Traumatol Surg Res*	Jul 2017–Jun 2018	France	Prospective cohort study	2	Circumferential repair	LBR
Sawyer^37^ (2015)	*Am J Sports Med*	2009-2011	USA	Prospective cohort study	3	Circumferential repair	LBR

a LBR, labral base refixation; LOE, level of evidence; NWB, nonweightbearing; WB, weightbearing.
